# Impact of Fixed-Duration Oral Targeted Therapies on the Economic Burden of Chronic Lymphocytic Leukemia in Canada

**DOI:** 10.3390/curroncol30050339

**Published:** 2023-04-24

**Authors:** Jean Lachaine, Kimberly Guinan, Andrew Aw, Versha Banerji, Isabelle Fleury, Carolyn Owen

**Affiliations:** 1 Faculty of Pharmacy, University of Montreal, Montreal, QC H3T 1J4, Canada; 2 PeriPharm Inc., Montreal, QC H2Y 2H4, Canada; 3 Ottawa Hospital, Ottawa, ON K1H 8L6, Canada; 4 CancerCare Manitoba Research Institute, Winnipeg, MB R3E 0V9, Canada; 5 Departments of Internal Medicine and Biochemistry & Medical Genetics, Rady Faculty of Health Sciences, Max Rady College of Medicine, University of Manitoba, Winnipeg, MB R3E 0W2, Canada; 6 Maisonneuve-Rosemont Hospital, Institut Universitaire Hémato-Oncologie Transplantation Cellulaire, Montreal University, Montreal, QC H1T 2M4, Canada; 7 Foothills Medical Centre, Calgary, AB T2N 2T9, Canada

**Keywords:** chronic lymphocytic leukemia, oral targeted therapy, fixed-treatment-duration therapy, economic burden, prevalence

## Abstract

Background: Continuous oral targeted therapies (OTT) represent a major economic burden on the Canadian healthcare system, due to their high cost and administration until disease progression/toxicity. The recent introduction of venetoclax-based fixed-duration combination therapies has the potential to reduce such costs. This study aims to estimate the prevalence and the cost of CLL in Canada with the introduction of fixed OTT. Methods: A state transition Markov model was developed and included five health states: watchful waiting, first-line treatment, relapsed/refractory treatment, and death. The number of CLL patients and total cost associated with CLL management in Canada for both continuous- and fixed-treatment-duration OTT were projected from 2020 to 2025. Costs included drug acquisition, follow-up/monitoring, adverse event, and palliative care. Results: The CLL prevalence in Canada is projected to increase from 15,512 to 19,517 between 2020 and 2025. Annual costs were projected at C$880.7 and C$703.1 million in 2025, for continuous and fixed OTT scenarios, respectively. Correspondingly, fixed OTT would provide a total cost reduction of C$213.8 million (5.94%) from 2020 to 2025, compared to continuous OTT. Conclusions: Fixed OTT is expected to result in major reductions in cost burden over the 5-year projection, compared to continuous OTT.

## 1. Introduction

Chronic lymphocytic leukemia (CLL) is the most common form of adult leukemia, representing 44% of all leukemia cases in Canada in 2016 [1]. CLL is often diagnosed in the elderly, with a mean age at diagnosis of 71 years. In most patients, CLL is initially managed through watchful waiting (WW), with treatment initiation required after a median time of 4.8 years of surveillance [2]. Although CLL treatments are non-curative, the recent development and availability of highly effective therapies has resulted in prolonged survivals.

The selection of treatment regimens and prognosis are influenced by the fitness and age of the patients, as well as mutation status. For example, mutations at the immunoglobulin heavy chain variable (*IGHV*) region gene are associated with more durable responses to chemoimmunotherapy (CIT) treatments, whereas deletion at chromosome 17p or *TP53* gene mutations [del(17p)] is associated with a poor prognosis and resistance to chemoimmunotherapy [3].

The development of oral targeted therapies (OTT) has led to substantial improvements in CLL treatments. Although CIT regimens were the standard of care for CLL patients for many years, continuous OTTs have emerged as the current standard of care for CLL in Canada. Ibrutinib, the first in class Bruton tyrosine kinase (BTK) inhibitor, was first reimbursed in 2015 for patients who had received at least one prior therapy and were considered inappropriate for treatment or re-treatment with a fludarabine-based regimen, and later reimbursed for treatment-naïve patients starting in 2016–2017 for patients considered inappropriate for fludarabine-based treatment due to high-risk disease such as patients with del(17p) and, in some provinces, for high-risk patients including those who are young and fit with unmutated *IGHV* status as of 2018 [4,5]. A second generation BTK inhibitor, acalabrutinib, was recently approved in Canada in 2019 and is now reimbursed by all provinces for previously untreated CLL as well as for CLL patients who have received at least one prior therapy [6,7]. These continuous OTTs have superior efficacy, easier administration, and reduced toxicity as compared to CIT [8]. In the first-line CLL RESONATE-2 trial, ibrutinib showed significant improvement over chlorambucil in progression-free survival (PFS) in patients with and without *IGHV* and without del(17p) mutations, with a 5-year PFS of 70%, compared to 12% in those treated with the chemotherapy comparator, chlorambucil [9]. Overall survival (OS) rate also increased with ibrutinib, irrespective of high-risk clinical or genomic features [9,10]. The median duration of ibrutinib treatment was reported as 57.1 months in first-line treatment and 41.0 months in second-line treatment, with some patients receiving treatment for more than 5–6 years [9,10]. Since these OTTs are administered continuously until disease progression or toxicity, and given their relatively high cost, continuous OTT represents a major economic burden on the Canadian healthcare system despite the improvements on survival and disease control for individual patients [11].

Venetoclax, marketed as a single agent in 2016, was recently approved by Health Canada and recommended for reimbursement by CADTH as part of two fixed-treatment-duration regimens: (1) in combination with rituximab (VR) for 24 months for the treatment of adult CLL patients who have received at least one prior therapy, and (2) in combination with obinutuzumab (VO) for 12 months for the treatment of patients with previously untreated CLL [12,13]. The VR indication received reimbursement in 2020 in all Canadian provinces, while the VO indication received reimbursement starting in late 2021 and is now funded in all provinces [12,13]. The efficacy of VO and VR demonstrated that after treatment cessation, the venetoclax-based treatment combination continues to significantly improve PFS compared with CIT [14,15,16]. VO demonstrated a 3-year PFS of 81.9% versus 49.5% in the chlorambucil plus obinutuzumab group 2 years following treatment cessation [14,15,16]. The introduction of venetoclax-based combinations administered as fixed-duration treatment offers targeted CLL treatment options that allow for better budget predictability and potential cost savings to the healthcare system. Thus, this study aims to estimate the cost burden of CLL in Canada with the introduction of fixed-treatment-duration OTT compared to current continuous OTT, while taking into account the future prevalence of the disease.

## 2. Materials and Methods

### 2.1. Model Structure

A previously published state transition Markov model, by Lachaine et al., was adapted to assess the introduction of fixed-treatment-duration OTT. The Markov model structure includes four health states (WW, first-line treatment, relapsed/refractory treatment, and death), as detailed in Lachaine et al. [11].

### 2.2. Patient Characteristics 

The patient characteristics and estimations made for the incidence and prevalence of CLL in the Canadian population have been previously described [11]. Briefly, patients were defined by age (<65, 65–70 or >70 years old), phase of CLL treatment (WW, first-line or relapsed/refractory) and fitness level as well as del(17p) or *IGHV* mutation. Model input parameters were updated with the most recent data available, as shown in Table 1.

Similar to Lachaine et al., a warm-up period was incorporated into the model to estimate the prevalent population living with CLL in 2020. The warm-up period was used to build the study population in 2020 with the appropriate proportion of patients at the different stages of their disease. The 2020 population was calculated by adding new incident cases over a 10-year period, from 2010 to 2020, into the model, stratified using clinical practices from this period. The annual incidence of CLL per year was calculated using data from Statistics Canada [17]. The total number of incident cases was calculated by applying the annual rate of CLL per patient to the total Canadian population on a yearly basis. After this warm-up period, the model generates a population reflecting 2016 epidemiological data. Since the prevalence could be overestimated due to PFS and OS clinical trial data overestimation compared to clinical practice (exclusion of co-morbidities, better stratification, patient ages, etc.), model calibration was carried out using real-world data (prevalence at a defined time, drug utilization, etc.) along with the Excel Solver function [11]. The model assumptions are presented in Table 1.

**Table 1:** Model and Costs ^a^ Parameters.

Parameters	Model	Reference
Probabilities, % (unless otherwise stated)		
Probability of WW at diagnosis	85.00	Assumption; Chen [18]
Median time to first treatment (years)	4.8	Parikh [2]
Transition probability from WW to first treatment by cycle	1.65	Model calibration
Proportion of patients in IV therapy	100.00	When both formulas available
Prevalence of del(17)p	7.00	Hallek [19]
Proportion of mutated IGHV	40.00	Assumption, confirmed by KOL
Proportion of non-mutated IGHV	60.00	Assumption, confirmed by KOL
Median age at diagnosis (years)	71	LLSC [1]
Probability of age at diagnosis		Statistics Canada [17]
Age < 65 years	33.75 *	
Age 65–70 years	14.34 *	
Age > 70 years	51.91 *	
Probability of fitness		Assumption, confirmed by KOL
Age < 65 years	90.00	
Age 65–70 years	50.00	
Age > 70 years	15.0 *	
Probability of discontinuing OTT for each 4-week cycle		Burger [20] Model calibration
In first-line treatment	0.70	
For relapsed patients	1.40	
Probability of death by cycle of 28 days according to the repartition of CLL age Category	0.695 *	Statistics Canada [21] Model calibration
Costs, C$		
Follow-up and laboratory monitoring costs		
Electrolyte panel	18.08 *	Code L226, 204, 053, 165, 194, 061, 700 [22]
Renal panel	13.32 *	Code L251, 067, 700 [22]
Liver function test	21.15 *	Code L223, 222, 191, 029, 030, 031, 005, 208, 700 [22]
CBC panel	14.74 *	Code L393, 700 [22]
Coagulation parameters	13.42 *	Code L445, 700 [22]
Serology	21.01 *	Code L319, 700 [22]
Chemotherapy infusion, administration, and management	105.15	Schedule of benefits. Code G359 [23]
Professional fees		
Consultation, Hematology	157.00	Schedule of Benefits. Code A615 [23]
Partial assessment, Hematology	38.05	Schedule of Benefits. Code A618 [23]
Nurse average wage (C$/min)	0.63 *	Statistic Canada [24]; Job Bank Canada, NOC 3012 [25]
Pharmacist average wage (C$/min)	0.87 *	Statistic Canada [24]; Job Bank Canada, NOC 3131 [25]
Adverse events		
Anemia	759.71 *	OCC, code D649 [26]. Assuming 2% managed inpatient
Neutropenia	523.23 *	OCC, code D700 [26]. Assuming 100% managed outpatient
Febrile neutropenia	10,918 *	OCC, code R508 [26]. Assuming 100% managed inpatient
Thrombocytopenia	441.86 *	OCC, code D696 [26]. Assuming 100% managed outpatient
Infection	1831 *	OCC, code A499/B349 [26]. Assuming 25% managed inpatient
Atrial fibrillation	1413 *	OCC, code I4890 [26]. Assuming 10% managed inpatient
Palliative care	9326 *	CIHI, Code 810. (Average all adult patients, Canada)

CBC, complete blood count; del(17p), deletion at chromosome 17p or TP53 gene mutations; IGHV, immunoglobulin heavy chain variable; IV, intravenous; KOL, key opinion Leader; LLSC, Leukemia and Lymphoma Society of Canada; OCC, Ontario Care Costing; OTT, oral targeted therapy; WW, watchful waiting. ^a^ All costs are shown in 2020 Canadian dollars. * Theses values have been added or updated from the previously published model with most recent data.

### 2.3. Simulated Clinical Pathway

Within the four health states (WW, first-line treatment, relapsed/refractory treatment, and death), patients could enter the model either in WW or in the first-line treatment. If patients failed to respond to first-line treatment, they entered the relapsed/refractory health state. After failure to respond to a second-line treatment, patients entered a sub-health state of relapsed/refractory, where their disease progressed but death has not yet occurred (palliative state). Patients in each of the health states could transition to death. Patients within the model cannot revert to previous health states.

The probabilities of health state transitions were estimated based on PFS and OS from pivotal clinical trials and all-cause mortality rates (Table 2). Trials were selected based on the best and most recent evidence available, including phase III trials, for each treatment regimen. PFS was used to estimate the transition from first-line treatment to relapse as well as the progression from the relapse (second-line treatment) state. OS was used to determine the transition to death of relapse patients who progressed on second-line treatment. All-cause mortality rates were used to determine the transition to death from WW, first-line treatment and relapse (patients responding to treatment only) health states. As previously stated, note that all parameters were calibrated to obtain a population reflective of real-world epidemiological data. All probabilities were adjusted to fit model cycles of 28 days.

### 2.4. Treatment Algorithms

The treatment algorithms for each CLL patient were defined by multiple factors such as the line of treatment, patient characteristics, mutations and year of treatment. Algorithms were based on the Alberta Clinical Guidelines and adapted based on the comments of clinical experts in CLL [45]. The selected treatments are reimbursed by most Canadian provinces and their entry within the treatment pattern occurs at the time of first expected reimbursement in a Canadian province. Compassionate use is not considered within this model since the objective is to capture the economic burden of CLL from the public health care system perspective.

**Figure 1:**
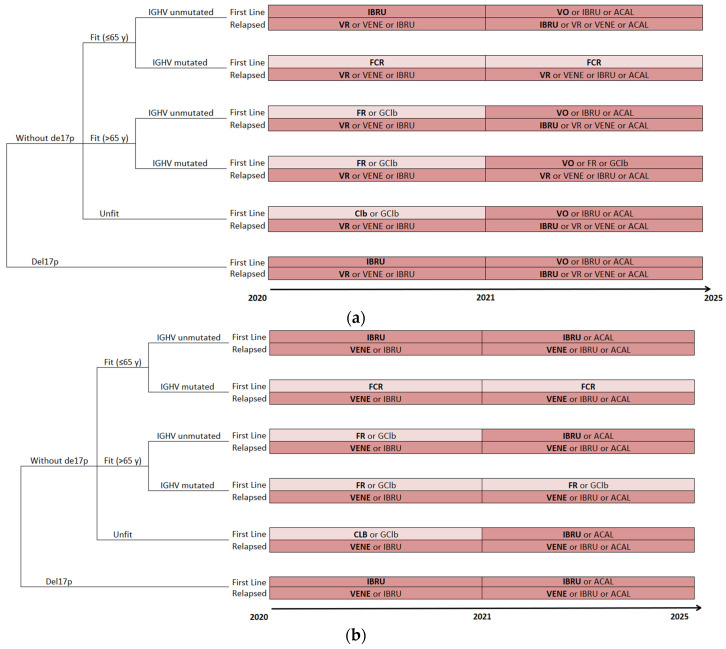
(**a**,**b**) Management strategies for CLL patients. Treatment algorithms of (**a**) fixed-treatment-duration oral targeted therapy (OTT) scenario and (**b**) continuous OTT scenario, with evolving therapeutic options between 2020–2025. The selected model treatments are presented in bold. ACAL, acalabrutinib; del(17p), deletion at chromosome 17p or *TP53* gene mutations; ACAL, acalabrutinib; FCR, fludarabine, cyclophosphamide and rituximab; FR, fludarabine and rituximab; GClb, obinutuzumab and chlorambucil; IBRU, ibrutinib; *IGHV*, immunoglobulin heavy-chain variable; R, rituximab; VENE, Venetoclax; VO: venetoclax and Obinutuzumab; VR: venetoclax and rituximab.

For the prevalent population, treatment algorithms were simulated from 2010 to 2020, which reflects the evolution of the standard of care and other therapies as well as changes in clinical practice, with the entry of continuous OTT. For the incident population, the treatment algorithms were simulated from 2020 to 2025, which reflects the evolution of the standard of care and other therapies, as well as changes in clinical practice, with the entry of fixed OTT (Figure 1a). An additional clinical scenario was considered, where continuous OTT would remain the standard of care in order to evaluate the impact of the introduction of fixed OTT as an alternative treatment option (Figure 1b).

### 2.5. Cost Data

To estimate the economic burden of CLL from a public healthcare perspective, only direct medical costs were considered, which includes costs associated with drug acquisition, follow-up/monitoring, adverse events (AEs) and palliative care (Table 1). Each cycle of treatment is 28 days. For drug acquisition costs, the unit cost of each treatment was obtained from IQVIA Delta PA (November 2020); similarly to the previously published model, treatment regimens were obtained from Cancer Care Ontario (CCO), and a body surface area of 1.89 m^2^ and a weight of 76 kg was used [11,46,47]. For continuous OTT, drug costs were accumulated until treatment discontinuation, either due to relapse or other clinical reasons. For fixed OTT, drug costs were accumulated until treatment completion or until treatment discontinuation, either due to relapse or other clinical reasons. A probability of discontinuation for each 28-day cycle was estimated at 0.70% and 1.40%, for first-line and relapse patients, respectively, as previously described in Lachaine et al [11,20].

The follow-up and monitoring costs consisted of those involved in laboratory tests, as well as administration and professional fees for nurses, pharmacists, and physicians. Routine laboratory tests are needed for all CLL patients under treatment or WW. The unit costs of laboratory tests were retrieved from the Schedule of Benefits for Laboratory Services from the Ontario Ministry of Health and Long-Term Care [22], while the testing frequency was obtained from CCO and validated by clinical experts in CLL. The costs for administration comprised the cost of chemotherapy infusion as well as professional fees, including the hematologist, nurse, and a pharmacist cost. The professional fee for the hematologist was retrieved from the Schedule of Benefits—Physician Services in Ontario, while the nursing and pharmacy workloads were determined by CCO and Statistics Canada, and their median wages obtained from Job Bank Canada [23,24,25]. The frequency of administrations per cycle was determined from CCO treatment regimens [46].

AEs were extracted from clinical trials and product monographs. The main AEs considered in the model include anemia, neutropenia, febrile neutropenia, thrombocytopenia, infection, and atrial fibrillation. Only grade 3 or 4 AEs were considered in this model. The cost per event was obtained from the Ontario Case Costing (OCC) analysis tool and the proportion of AE-managed inpatient or outpatient was determined by clinical experts in CLL [26].

Costs of palliative care were obtained from the Canadian Institute for Health Information (CIHI) patient cost estimator [48].

### 2.6. Model Outcomes

The number of CLL patients as well as total costs associated with CLL management in Canada were projected from 2020 to 2025. The total annual costs for first- and second-line treatments were calculated for both continuous- and fixed-treatment-duration OTT. All costs were converted to 2020 Canadian dollars and presented in rounded values to simplify comprehension.

### 2.7. Sensitivity Analysis

One-way sensitivity analyses (OWSA) were performed to assess the robustness of the model results. Since PFS, OS, and probability of discontinuation were directly varied through model calibration, they were not included in the sensitivity analyses. All other model parameters were varied with a range of ±25%.

## 3. Results

### 3.1. Disease Burden

**Figure 2:**
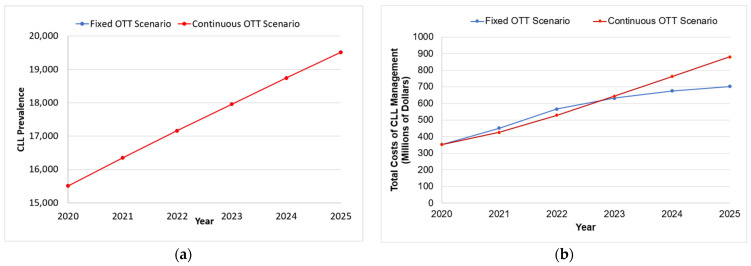
Trends in the disease and cost ^a^ burden of CLL under the fixed and continuous OTT scenarios. (**a**) The annual prevalence of CLL (including patients in the watchful waiting state) under fixed and continuous OTT scenarios. The use of fixed-treatment-duration OTT is projected to increase the number of patients living with CLL from 15,512 in 2020 to 19,517 by 2025, whereas patient prevalence will be increased to 19,513 in the continuous OTT scenario. (**b**) The total cost of CLL management per year under the fixed and continuous OTT scenarios. The use of continuous OTT is projected to increase the annual cost of CLL management from C$352.5 million to C$880.7 million, while the implementation of fixed OTT will only increase the cost to C$703.1 million. ^a^ All costs are shown in 2020 Canadian dollars.

As rates of incidence and survival are both increasing, the prevalence of CLL in Canada is projected to increase 1.3-fold from 15,512 patients in 2020 to 19,517 or 19,513 patients in fixed or continuous OTT scenarios, respectively (Figure 2a).

### 3.2. Cost Burden

#### 3.2.1. Total Annual Cost of CLL

Under the continuous OTT scenario, the total annual cost of CLL management is projected to increase from C$352.5 million in 2020 to C$880.7 million in 2025, a 2.5-fold increase (Figure 2b). The steady increase in cost reflects the costs of continuous treatment duration OTT as well as the period between 2021 to 2025 when ibrutinib becomes available for all patients except for those with mutated *IGHV* in first-line treatment. In comparison, the fixed OTT scenario is projected to increase 2.0-fold from C$352.6 million to C$703.1 million during the same time period. Although initially, the addition of treatment with the combination of venetoclax and rituximab during 2020, as well as the combination of venetoclax and obinutuzumab starting from 2021, generated a sharper increase in cost than the continuous OTT scenario, the cost of fixed OTT starts to plateau in the year 2023, once patients complete their fixed-duration treatment. The total cumulative costs over the projected 5-year period were estimated at C$3.596 billion for the continuous OTT scenario and C$3.382 billion for the fixed OTT scenario. Consequently, the fixed OTT scenario represents a total cost reduction of C$213.8 million (5.94%) from 2020 to 2025, compared to the continuous OTT scenario.

#### 3.2.2. Cost of First-Line Therapy for CLL

In first-line treatment, the cost of CLL treatment in the continuous OTT scenario is projected to increase from C$256.8 million to C$705.9 million between 2020 and 2025. In contrast, the cost in the fixed OTT scenario is projected to increase to C$536.5 million by 2025. The total costs in first-line treatment over the projected 5-year period was estimated at C$2.735 billion for the continuous OTT scenario and C$2.540 billion for the fixed OTT scenario. Consequently, the fixed OTT scenario represents savings of 7.15% (C$195.7 million) compared to the continuous OTT scenario.

#### 3.2.3. Cost of Second-Line Therapy for CLL

In second-line treatment, the cost of CLL treatment in the continuous OTT scenario is projected to increase from C$93.9 million to C$172.2 million from 2020 to 2025. In contrast, the cost in the fixed OTT scenario is projected to increase to C$164.1 million in 2025. The costs of second-line treatment over the projected 5-year period was estimated at C$847.0 for the continuous OTT scenario and C$829.2 billion for the fixed OTT scenario. Thus, costs are decreased by 2.10% (C$17.8 million) under the fixed OTT scenario.

### 3.3. Sensitivity Analysis

**Figure 3:**
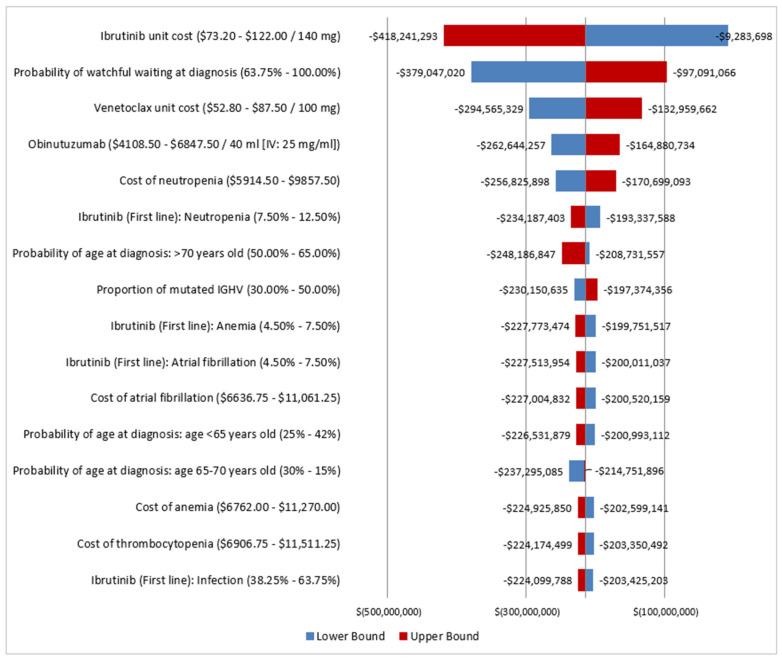
Sensitivity analysis of the difference in total annual costs of CLL between the fixed OTT and the continuous OTT scenarios. Tornado diagram for the one-way sensitivity analysis of the difference in total annual cost between the fixed OTT and the continuous OTT scenarios. CLL, chronic lymphocytic leukemia; del(17p), deletion at chromosome 17p or *TP53* gene mutations; *IGHV*, immunoglobulin heavy-chain variable; OTT: oral targeted therapy. All Costs are shown in 2020 Canadian dollars.

The OWSA showed that the cost of CLL management was most sensitive to the cost of ibrutinib, probability of watchful waiting at diagnosis, cost of venetoclax, cost of obinutuzumab, as well as neutropenia. The tornado diagram presenting the difference in total costs of CLL management from 2020 to 2025 between the fixed OTT and continuous OTT scenarios is presented in Figure 3.

## 4. Discussion

This study provides the first estimate of the economic burden related to CLL management with the launch of fixed-treatment-duration OTT in Canada. Although continuous OTT is currently the standard of care for CLL patients, fixed OTT, which was recently introduced to the Canadian healthcare system, has an added benefit of considerably reducing the cost burden for the treatment of CLL patients over the 5-year projected period. Our study estimates that from 2020 to 2025, there will be a 1.3-fold increase in the number of people living with CLL because of the increased disease incidence as well as improved survival under OTT treatment. Concurrently, the overall cost of CLL management in the continuous OTT scenario is predicted to increase to C$880.7 million by 2025, a 2.5-fold increase from 2020. The overall cost is predicted to reduce to C$703.1 million with the availability and adoption of fixed-treatment-duration OTT. It is however unknown how retreatment with finite OTT will impact the costs as the studies did allow for retreatment, and how the costs would be combined with continuous OTT once patients no longer respond to finite treatments. In the meantime, in alignment with patient values for tolerable, safe treatment options, implementation of fixed OTT could alleviate the financial burden associated with CLL treatment.

In a previously published study comparing the cost burden of continuous OTT to traditional CIT treatment, the annual cost of CLL management in 2025 was estimated to increase to C$957.5 million with the adoption of continuous OTT, representing a 15.7-fold increase in costs compared to the CIT scenario. This current study extends the comparison between continuous OTT and fixed-treatment-duration OTT, with total costs of continuous OTT in 2025 estimated at C$880.7 million. For this current study, most model parameters from our previous publication have remained similar, except for the inclusion of new treatments, recent clinical trial data and cost updates. In line with these modifications, results for continuous OTT are within the same price range and thus, demonstrate the robustness of the model.

Although the economic burden of fixed OTT in CLL has not been otherwise conducted, budgetary impact analyses (BIA) have been performed for VO and VR for a fixed treatment duration of 12 and 24 months, respectively, in the United States (US) and France [49,50,51]. In the US, the implementation of VO results in a cost saving of US$1.6 million per 1 million members under the US health plan over a 3-year time horizon [50]. Similarly, VR resulted in a cost saving of US $0.7 million [49]. In France, a BIA analysis suggested that although there is an increase in total cost when the combination of VO is implemented during year 1, it is followed by a cost saving in years 2 through 10, compared to other CLL treatments. Over the ten-year time horizon, the fixed-duration treatments allowed a total cost saving of EUR $860 million [51]. Therefore, although these studies are not directly comparable to our economic burden study, the conclusion is consistent that implementing fixed-treatment-duration OTT can potentially alleviate economic burden on the healthcare system.

Our analysis has several limitations. First, patients receiving treatment in the model can only receive first- or second-line treatment. However, this does not necessarily reflect the clinical setting, as patients can also receive third and subsequent-line treatments. Although this option is excluded in our analysis, the costs included in this study reflect our objective of comparing the cost of continuous versus fixed OTT. Additionally, a fixed rate of CLL patients with del(17p) mutation was assumed for both first- and second-line treatments. However, the percentage of patients with del(17p) mutation can increase to more than 30% over time, particularly in relapsed patients [52]. Furthermore, the hospitalization due to dose ramp up of venetoclax was not considered in our model, though other cost factors of laboratory testing and nursing/physician assessments were included. However, according to the DEVOTE study, an observational study of relapsed/refractory CLL patients initiating VR or venetoclax in routine clinical practice in Canada, the utilization of hospitalization due to dose ramp up varies greatly across Canada [53]. According to study results, approximately one third of patients were hospitalized at venetoclax initiation (median duration of 2 days), and venetoclax hospitalizations for subsequent dose ramp ups were even less frequent. Therefore, the exclusion of this cost parameter is not expected to greatly impact the results of the study. The use of obinutuzumab in combination with venetoclax may increase costs due to the nature of the infusion and the time it takes to deliver it. There may also be associated drug costs and hospitalizations that are not accounted for.

Lastly, to manage toxicities and adverse events associated with OTT, dose reduction could be required for patients on ibrutinib and venetoclax [54,55,56,57]. As data supporting the clinical efficacy of dose reduction are largely from real world experience, they are observational and need to be formally studied to establish definitive clinical practice guidelines [56,58,59]. Therefore, dose reductions for either fixed or continuous OTT were not considered in this study. However, it is possible that use of lower doses in clinical practice due to AE management can lower the economic burden in both continuous and fixed OTT scenarios in the future. To partially assess this limitation, a sensitivity analysis varying the unit cost of ibrutinib and venetoclax by ±25% was performed; both upper and lower bound results showed a reduction in the economic burden. In addition, it is also possible that the rates of discontinuation are overestimated as they were derived based on the rates of previous chemotherapies. In the literature, the rates of discontinuation of OTT range between 15–20%; however this data is still emerging [20].

## 5. Conclusions

In conclusion, this study highlights that although prevalence and cost associated with CLL in Canada are predicted to increase, the introduction of fixed-treatment-duration OTT will lead to a reduction in the cost burden of CLL treatment. Changes in pricing and clinical practices, such as dose reduction for adverse event management or more targeted and personalized treatment regimens, may further help alleviate the economic burden of CLL management.

## Figures and Tables

**Table 2:** Summary of Treatment-Related Parameters.

Treatments	PFS and OS	Grade 3 or 4 Adverse Events (%)	Drug Cost ^a^ (C$/Cycle)	References
**First-line setting**				
GClb	Median PFS, 29.8 months	Anemia, 4 Neutropenia, 33 Thrombocytopenia, 10 Infection, 12	Cycle 1: 16,497 Cycles 2–6: 5541	Goede [27] Goede, 2014 [28]
FCR	<65 years, 5-year PFS, 48% ≥65 years, 5-year PFS, 43% IGHV mutated, 5-year PFS, 67% IGHV unmutated, 5-year PFS, 33%	Anemia, 6 Neutropenia, 30 Thrombocytopenia, 9 Infection, 24	Cycle 1: 3067 Cycles 2–6: 3769	Fischer [29] Hallek [19]
FR	Median PFS, 42.0 months	Anemia, 40 Neutropenia, 76 Thrombocytopenia, 20 Infection, 20	Cycle 1: 3194 Cycles 2–6: 3896	Woyach [30] Byrd [31]
F	Median PFS, 19.0 months	Anemia, 15 Neutropenia, 12 Thrombocytopenia, 15 Infection, 80	1089	Eichhorst [32]
BR	>70 years, median PFS, 43.0 months	Anemia, 31 Neutropenia, 31 Thrombocytopenia, 35 Infection, 12	Cycle 1: 6357 Cycles 2–6: 7059	Woyach [33] Fischer [34]
Clb	Median PFS, 15.0 months	Anemia, 27 Neutropenia, 12 Thrombocytopenia, 20 Infection, 4	Cycle 1: 264.30 Cycles 2–6: 176.20	Burger [9] Eichhorst [32]
Ibrutinib	5-year PFS, 70% TP53 mutation, 5-year PFS, 56% IGHV mutated, 5-year PFS, 81% IGHV unmutated, 5-year PFS, 67%	Anemia, 6 Neutropenia, 10 Thrombocytopenia, 2 Infection, 6 Atrial fibrillation, 6	8198	Burger [9,20]
ACAL	24-month PFS, 87%	Anemia, 7 Neutropenia, 10 Thrombocytopenia, 5 Infection, 5	7615	Sharman [35]
VO	3-year PFS, 82% Del(17p), 3-year PFS, 49% IGHV mutated, 3-year PFS, 87% IGHV unmutated, 3-year PFS, 81%	Anemia, 8 Neutropenia, 53 Febrile neutropenia, 5 Thrombocytopenia, 14 Infection, 18	Cycle 1: 16,532 Cycle 2: 9153 Cycles 3–6: 13,318 Cycles 7–13: 7840	Al-Sawaf [14] Fischer [36]
**Second-line setting**				
F	Median PFS, 14.8 months Median OS, 41.0 months	Anemia, 80 Neutropenia, 17 Thrombocytopenia, 60 Infection, 15	1089	Niederle [37]
FCR	Median PFS, 28.0 months Median OS, 42.0 months	Anemia, 24 Neutropenia, 81 Thrombocytopenia, 34 Infection, 16	Cycle 1: 3067 Cycles 2–6: 3769	Wierda [38]
Ibrutinib	Median PFS, 44.1 months Del (17p) median PFS, 40.6 months IGHV mutated, median PFS, 48.4 months IGHV unmutated, median PFS, 49.7 months Median OS, 67.7 months Del(17p) median OS, 61.8 months	Anemia, 0 Neutropenia, 18 Thrombocytopenia, 10 Infection, 51 Atrial fibrillation, 6	8198	Munir [10] Byrd [39]
BR	2-year PFS, excluding del(17p), 17% 5-year OS, 62%	Anemia, 14 Neutropenia, 39 Thrombocytopenia, 10 Infection, 22	Cycle 1: 6357 Cycles 2–6: 7059	Kater [40] Seymour [15]
ACAL	12-month PFS, 88% 12-month OS, 94%	Anemia, 7 Neutropenia, 14 Febrile neutropenia, 2 Thrombocytopenia, 2 Infection, 1 Atrial fibrillation, 2	7615	Ghia [41] Byrd [42]
Venetoclax	Median PFS, 24.7 months Del (17p), 24-month PFS, 54% 12-month OS, 91% Del (17p), 24-month OS, 73%	Anemia, 29 Neutropenia, 51 Febrile neutropenia, 13 Thrombocytopenia, 29 Infection, 11	Cycle 1: 1813 Cycles 2+: 7840	Jones [43] Stilgenbauer [44]
VR	Median PFS, 53.6 months Without del(17p), median PFS, 56.6 months Del(17p), median PFS, 45.3 months <65 years, median PFS, 49.0 months ≥65 years, median PFS, 57.0 months 5-year OS, 82%	Anemia, 11 Neutropenia, 58 Febrile neutropenia, 4 Thrombocytopenia, 6 Infection, 18	Ramp-up: 3773 Cycle 1: 9945 Cycles 2–6: 10,647 Cycles 7+: 7840	EMA [16] Seymour [15]

ACAL, acalabrutinib; BR, bendamustine and rituximab; Clb, chlorambucil; del(17p), deletion at chromosome 17p or TP53 gene mutations; F, fludarabine; FCR, fludarabine, cyclophosphamide and rituximab; FR, fludarabine and rituximab; GClb, obinutuzumab and chlorambucil; IGHV, immunoglobulin heavy-chain variable; OS, overall survival; PFS, progression-free survival; R, rituximab; VO, Venetoclax and Obinutuzumab; VR, venetoclax and rituximab;. ^a^ All Costs are shown in 2020 Canadian dollars.

## Data Availability

The data presented in this study are available within this article.
